# Angiotensin-Converting Enzyme Inhibitors (ACEIs) and Angiotensin-Receptor Blockers (ARBs) in Patients at High Risk of Cardiovascular Events: A Meta-Analysis of 10 Randomised Placebo-Controlled Trials

**DOI:** 10.1155/2013/478597

**Published:** 2013-11-06

**Authors:** Hean Teik Ong, Loke Meng Ong, Jacqueline Judith Ho

**Affiliations:** ^1^Consultant Cardiologist, HT Ong Heart Clinic, 251C Burma Road, Penang 10350, Malaysia; ^2^Consultant Nephrologist and Head, Department of Medicine and Clinical Research Centre, Penang Hospital, Penang 10990, Malaysia; ^3^Clinical Epidemiologist, Professor and Head of Paediatrics, Penang Medical College, Penang 10450, Malaysia

## Abstract

*Context*. Whether angiotensin converting-enzyme inhibitors (ACEI) and angiotensin-receptor blockers (ARB) are useful in high risk patients without heart failure is unclear. We perform a meta-analysis of prospective randomized placebo-controlled ACEI or ARB trials studying patients with a combination of risk factors to assess treatment impact on all cause mortality, cardiovascular mortality, nonfatal myocardial infarction (MI) and stroke. *Method*. A PubMed search was made for placebo-controlled trials recruiting at least 1,200 high risk patients randomized to either ACEI or ARB, with follow-up of at least 2 years. Meta-analysis was performed using the RevMan 5 program and Mantel-Haenszel analysis was done with a fixed effects model. *Results*. Ten trials recruiting 77,633 patients were reviewed. All cause mortality was significantly reduced by ACEI (RR 0.89; *P* = 0.0008), but not by ARB treatment (RR 1.00; *P* = 0.89). Cardiovascular mortality and nonfatal MI were also reduced in the ACEI trials but not with ARB therapy. Stroke was significantly reduced in the ACEI trials (RR 0.75; *P* < 0.00001) and more modestly reduced in the ARB trials (RR 0.90; *P* = 0.01). *Conclusion*. ACEI treatment reduced stroke, nonfatal MI, cardiovascular and total mortality in high risk patients, while ARB modestly reduced stroke with no effect on nonfatal MI, cardiovascular and total mortality.

## 1. Introduction

Angiotensin-converting enzyme inhibitors (ACEIs) and angiotensin-receptor blockers (ARBs) have been shown to reduce cardiovascular outcomes in patients with heart failure or hypertension [1–3]. However, whether ACEI and ARB are useful in reducing cardiovascular events amongst patients at risk from a variety of clinical conditions but without left ventricular systolic dysfunction is more debatable. Several meta-analyses have addressed this issue, but these solely reviewed either ACEI or ARB alone or looked at patients with a single disease condition like hypertension or ischemic heart disease [4–10]. Both ACEI and ARB produce inhibition of the rennin-angiotensin system and have been shown to be equivalent in their blood pressure lowering effect [11]. We thus seek to answer the question of whether ACEI and ARB are useful and equivalent in their reduction of total mortality, cardiovascular mortality, nonfatal myocardial infarction (MI), and stroke in patients with normal systolic function and who are at high risk of cardiovascular events from a combination of various clinical conditions. 

## 2. Methods

This present meta-analysis seeks to address the question of whether ACEI and ARB should be routinely used in patients at high risk of adverse cardiovascular events; the Heart Outcomes Prevention Evaluation Study (HOPE) is the pioneering trial addressing this subject [12]. High-risk patients are those with a combination of cardiovascular risk factors such as hypertension, diabetes, dyslipidemia, or presence of prior clinical atheromatous condition such as coronary, cerebrovascular, or peripheral arterial disease. We excluded trials in which patients were recruited based on the presence of a specific disease condition—hypertension, heart failure, diabetes, or acute MI—as the usefulness of ACEI or ARB in a single condition is not the subject we are presently investigating; the antihypertensive and lipid-lowering treatment to prevent heart attack trial (ALLHAT) which is a study of hypertension exemplifies the type of trials that we wish to exclude [13]. We only included trials where the end-points studied were clinical cardiovascular outcomes. We omitted ONTARGET and similar trials which compared ACEI treatment with ARB and combination therapy. These are not placebo-controlled studies similar to our other trials analysed but actually compared different treatment strategies with each other.

**Figure 1:**
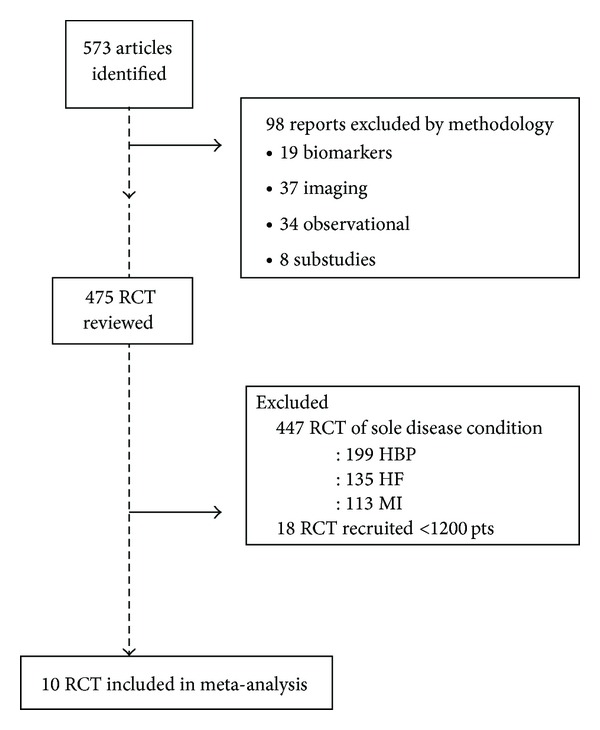
Search strategy. RCT: randomized controlled trials; HBP: hypertension; HF: heart failure; MI: myocardial infarction; pts: patients.

A PubMed search was conducted for trials published from 1990, first using the search terms “Angiotensin Receptor Antagonists” OR “Angiotensin-Converting Enzyme Inhibitors” followed by using “coronary artery disease” OR “cardiovascular disease” OR “coronary angioplasty” OR “stroke” OR “transient ischemic attack” OR “TIA” OR “peripheral vascular disease” OR “high risk” followed by “cardiovascular event” OR “cardiovascular death” OR “cardiovascular mortality” OR “myocardial infarction” OR “death” OR “mortality” OR “total mortality”. A total of 573 publications were identified. We excluded observational trials, substudy reports, or studies primarily involving biomarkers or imaging modalities. We found 475 prospective randomized controlled trials with clinical end-points. We selected prospective, randomized, placebo-controlled clinical trials recruiting high-risk patients involving at least 1200 patients followed-up for at least 2 years and excluded trials studying patients with a single specific condition or risk factor such as hypertension, diabetes, or heart failure as the usefulness of ACEI or ARB in each of these specific conditions is not the subject we presently seek to investigate (Figure 1). 

Ten trials fulfilled our inclusion and exclusion criteria. The trials were assessed for risk of bias based on the presence or absence allocation concealment, blinding of the participant, care-giver, researcher, and outcome assessor, loss to follow up of <5%, and use of intention to treat analysis. Outcomes were independently extracted from the trials using a specially designed data extraction form. All authors were involved in data analysis and write up. 

Meta-analysis was performed using the RevMan 5 program [14]. Mantel-Haenszel analysis was done using a fixed effects model. We analyzed the effect of treatment on all-cause mortality, cardiovascular mortality, nonfatal MI, and stroke. Results were expressed as relative risks (RR) and 95% confidence intervals (CI). Three RRs were calculated for each outcome, one for all trials and one each for trials involving ACEI and trials involving ARB. We used the *I*-squared statistical test to explore for heterogeneity. Where heterogeneity was found we attempted to explain this clinically. We intended to use a random effects model if *I*-square was greater than 60%. 

## 3. Results

Ten trials—HOPE, PROGRESS, QUIET, EUROPA, CAMELOT, PEACE, JIKEI, TRANSEND, PROFESS and NAVIGATOR—were included in this meta-analysis [12, 15–23]. All trials were prospective, randomized, placebo-controlled trials. All had adequate allocation concealment, were blinded to participant, researcher, caregiver, and outcome assessor, and all were analyzed on an intention to treat basis. Less than 1% of those enrolled were lost to followup or withdrew after randomization in all trials reviewed except in PEACE, where 1.6% of patients were lost to followup.

The baseline characteristics of patients in the trials are as shown in Table 1. A total of 77,633 patients were enrolled in the 10 trials, with mean followup ranging from 2 to 5 years. Half of the recruited patients were in trails comparing ACEI with placebo (38988 patients; 50.2%), with the remainder involved in trials comparing ARB with placebo (38645 patients; 49.8%). The recruited patients were at high risk of cardiovascular outcomes because of prior atheromatous disease (coronary, cerebrovascular, or peripheral arterial) or else had multiple risk factors for cardiovascular ischemic events. The proportion of trial patients with diabetes varied from 0% to 38%, while the proportion with hypertension ranged from 27% to 88% (Table 1). 

### 3.1. All-Cause Mortality

**Figure 2:**
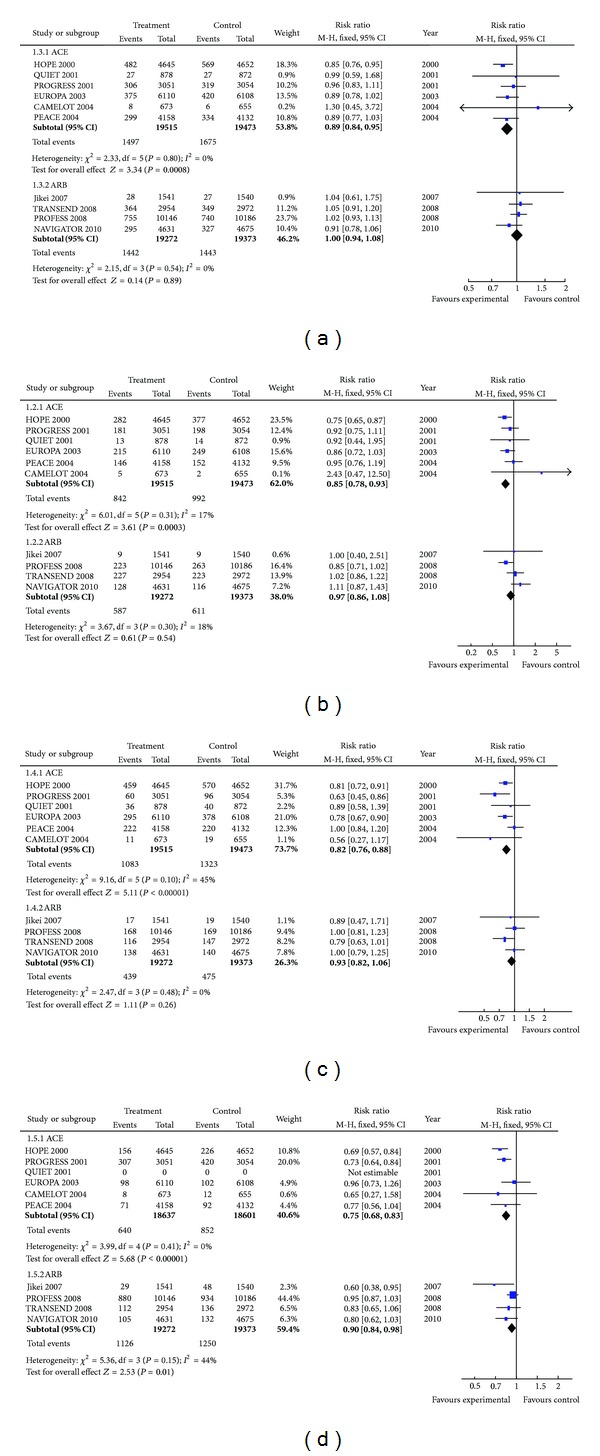
(a) Total mortality. (b) Cardiovascular mortality. (c) Nonfatal myocardial infarction. (d) Total stroke. HOPE: heart outcomes prevention evaluation; PROGRESS: perindopril protection against recurrent stroke study; QUIET: quinapril ischemic event trial; EUROPA: European trial on reduction of cardiac events with perindopril in stable coronary artery disease; CAMELOT: comparison of amlodipine versus enalapril to limit occurrences of thrombosis; PEACE: prevention of events with angiotensin converting enzyme inhibitors; JIKEI: valsartan in a Japanese population with hypertension and other cardiovascular disease; TRANSCEND: telmisartan randomized assessment study in ACE-intolerant subjects with cardiovascular disease; PROFESS: telmisartan to prevent recurrent stroke and cardiovascular events; NAVIGATOR: nateglinide and valsartan in impaired glucose tolerance outcomes research. HOPE [12] PROGRESS [15] QUIET [16] EUROPA [17] CAMELOT [18] PEACE [19] JIKEI [20] TRANSCEND [21] PROFESS [22] NAVIGATOR [23].

All-cause mortality was significantly reduced in trials comparing ACEI with placebo (7.67% versus 8.6%; RR 0.89, 95% CI 0.84–0.95; *P* = 0.0008) but was not significantly changed in the ARB-placebo trials (7.48% versus 7.45%; RR 1.00, 0.94–1.08; *P* = 0.89). No heterogeneity was noted in either the ACEI or ARB trials included in this meta-analysis. Thus, ACEI but not ARB appears to reduce total mortality in high-risk patients (Figure 2(a)).

### 3.2. Cardiovascular Mortality

Cardiovascular mortality was significantly reduced in the ACEI-placebo trials (4.31% versus 5.09%; RR 0.85, 0.78–0.93; *P* = 0.0003) but was not significantly affected by ARB treatment (3.05% versus 3.15%; RR 0.97, 0.86–1.08; *P* = 0.54). There was no heterogeneity in each group of trials analyzed. In patients at high risk, ACEI but not ARB significantly reduced cardiovascular mortality (Figure 2(b)).

### 3.3. Nonfatal MI

Compared to placebo, ACEI treatment significantly reduced nonfatal MI in patients at high risk (5.55% versus 6.79%; RR 0.82, 0.76–0.88; *P* < 0.00001). ARB therapy did not affect incidence of nonfatal MI (2.28% versus 2.45%; RR 0.93, 0.82–1.06; *P* = 0.26). No heterogeneity was noted within the ACEI and ARB trials. In patients at high risk, ACEI but not ARB significantly reduced nonfatal MI (Figure 2(c)).

### 3.4. Stroke

Stroke was significantly reduced in the ACEI-placebo trials (3.43% versus 4.58%; RR 0.75, 0.68–0.83; *P* < 0.00001) and to a lesser but still significant degree in the ARB-placebo trials (5.84% versus 6.45%; RR 0.90, 0.84–98; *P* = 0.01). No heterogenicity was noted within ACEI trials but there was modest heterogeneity in the ARB trials. This is because the definition of cerebrovascular event in JIKEI included transient ischemic attacks, unlike in the other trials [20]. This heterogeneity disappeared when the JEKEI study was excluded, although there was no substantial change in the RR (0.90 with and 0.92 without JEKEI). Thus, both ACEI and ARB reduce stroke incidence, although the effect from ACEI is greater (Figure 2(d)).

## 4. Discussion

It is important to appreciate that, despite overlapping patient characteristics, the trials selected are different from the studies of hypertension or those recruiting patients all having a specific disease or risk factor. Our target patient at high risk of cardiovascular events can have a combination of clinical conditions and risk factors but not all will have a particular condition like hypertension or dyslipidemia. Studying high-risk patients as a specific group was a novel idea until the HOPE trial. There was in fact much debate that the positive results from HOPE were due to the BP lowering effect of ramipril [24, 25]. The fact that less than 50% of patients in HOPE had hypertension argues against the benefit coming solely from hypertension control. We feel there is a need to distinguish such high-risk patients as recruited in HOPE from those recruited into hypertensive or dyslipidemic or diabetic trials, which are designed to gather information about management of a specific disease condition. In seeking to answer the question of whether ACEI or ARB therapy is able to reduce adverse cardiovascular outcomes in patients at high risk, it is important that we analyse only the prospective, randomised, placebo-controlled trials that actually address this issue. Thus, we excluded ONTARGET and similar trials that had no placebo arm but compared active ACEI therapy with ARB or their combination. These trials are a comparison of different strategies of rennin-antagonism and do not answer the question we are addressing.

Our meta-analysis has shown that ACEI and ARB are not equivalent in their effect on clinical outcomes. In high-risk patients, compared to placebo, ACEI treatment significantly reduced total mortality, cardiovascular mortality, nonfatal MI, and stroke. Our meta-analysis also shows that in high-risk patients, when compared to placebo, ARB treatment has no significant effect on cardiovascular or total mortality, as well as nonfatal MI. Calculation of the needed to treat (NNT) allows a comparison of the clinical impact of ACEI with ARB in stroke reduction. The small benefit from ARB (5.84% versus 6.45%; NNT 164) in reducing stroke is less pronounced than the effect obtained from ACEI therapy (3.43% versus 4.58%; NNT 87). It thus appears that the ARB is inferior to the ACEI and cannot be considered its therapeutic alternative when contemplating reduction of adverse cardiovascular clinical outcomes in the high-risk group. This contrasts with the situation in heart failure where the ARB is interchangeable with ACEI [2]. Thus, in patients at high risk of coronary events, ACEI should be offered before ARB, which should be used only in those intolerant of ACEI. 

However, the benefit of ACEI in reducing clinical cardiovascular events in the high-risk group is modest. The NNT to prevent total death is 108, to prevent cardiovascular death is 128, to prevent a nonfatal MI is 81, and to prevent stroke is 87. The NNT to prevent total mortality is 42 with beta-blockers after MI and is between 25–56 with simvastatin in the secondary prevention trials [26–28]. ACEI are more effective in preventing mortality in patients with impaired LV function; the NNT for total mortality is 22 using enalapril in patients with heart failure and an EF below 35% [1]. Thus, while ACEI are first line drugs in patients with heart failure and poor LV function, our data suggest they should be used only after statins and beta-blockers when seeking to reduce mortality and adverse clinical events in those with normal systolic function at high cardiovascular risk.

It is important in pooling studies that the statistical methodology employed does not lead to incorrect results, and authors do not produce overall conclusions that are inconsistent with the individual data [25, 26]. In our meta-analysis, the trials pooled together did not exhibit any heterogeneity, allowing greater confidence in pooling them together and in the validity of the overall findings. ACEI and ARB have been compared to other agents in patients with poor systolic function, hypertension, and diabetic nephropathy [2, 27–37]. We intentionally omitted such trials from our analysis as these patients are different from the group we have addressed, which are patients with high risk for or with past history of clinical atheromatous disease. The 95% confidence interval (CI) for most of the trials analyzed is narrow. In the ACEI-placebo trials, the midpoint of the CI in most of the individual studies point to a benefit from ACEI therapy agreeing with the pooled conclusion; in the ARB trials, the individual midpoints are very close to 1 and thus are in agreement at the absence of any treatment effect. The confidence in the correctness of our conclusion is thus enhanced. In patients at high risk of clinical atheromatous disease, these two groups of drugs do not have the same effect. ACEI reduced stroke, nonfatal MI, and cardiovascular and total mortality in high-risk patients, while ARB only modestly reduced stroke and has no effect on nonfatal MI, cardiovascular, and total mortality.

## Figures and Tables

**Table 1:** Baseline characteristic of patients in trials.

Trial	HOPE	PROGRESS	QUIET	EUROPA	CAMELOT	PEACE	JIKEI	TRANSCEND	PROFESS	NAVIGATOR
Year	2000	2001	2001	2003	2004	2004	2007	2008	2008	2010
Patient type	High risk	CVD	CAD	CAD	CAD	CAD	High risk	High risk	CVD	High risk
Number	9297	6105	1750	12218	1991	8290	3081	5926	20332	9306
FU (yr)	5	3.9	2.3	4.2	2	4.8	3.1	4.7	2.5	5.0
ACE/ARB dose	rami 10	perin 8	quina 20	perin 8	ena 20	tran 4	val 40–160	tel 80	tel 80	val 80–160
Mean age (yr)	66	64	58	60	58	64	65	67	66	64
BP (mmHg)	139/79	147/86	123/74	137/82	129/78	133/78	139/81	141/82	144/84	140/83
BMI	28	NS	NS	NS	30	NS	24	28	27	31
Sex (F %)	27	30	18	15	26	18	34	43	36	51
% CAD	80	16	100	100	100	100	34	75	NS	24
% CVD	11	100	NS	3	4	7	NS	22	100	3
% PAD	44	NS	NS	7	NS	NS	NS	11	NS	3
% DM	38	12	16	12	18	17	20	36	28	0
% HBP	47	48	47	27	60	46	88	76	74	78
% Smoking	14	20	22	NS	26	15	17	10	21	11

Yr: year; F: female; ACE: angiotensin converting enzyme; ARB: angiotensin receptor blocker; CAD: coronary artery disease; CVD: cerebrovascular disease; PAD: peripheral arterial disease; DM: diabetes mellitus; HBP: hypertension; NS: not significant; rami: ramipril; perin: perindopril; quina: quinapril; ena: enalapril; tran: trandolapril; val: valsartan; tel: telmisartan.

HOPE: heart outcomes prevention evaluation; PROGRESS: perindopril protection against recurrent stroke study; QUIET: quinapril ischemic event trial; EUROPA: European trial on reduction of cardiac events with perindopril in stable coronary artery disease; CAMELOT: comparison of amlodipine versus enalapril to limit occurrences of thrombosis; PEACE: prevention of events with angiotensin converting enzyme inhibitors; JIKEI: valsartan in a Japanese population with hypertension and other cardiovascular disease; TRANSCEND: telmisartan randomized assessment study in ace-intolerant subjects with cardiovascular disease; PROFESS: telmisartan to prevent recurrent stroke and cardiovascular events; NAVIGATOR: nateglinide and valsartan in impaired glucose tolerance outcomes research.
